# STAT1-mediated interferon signaling in the hematopoietic system is essential for restricting Usutu virus infection *in vivo*

**DOI:** 10.1371/journal.pntd.0013317

**Published:** 2025-07-22

**Authors:** Amy N. Nelson, Saloni Sinha, Sydney J. Mullin, Sebastian S. Carver, Thomas R. Cafiero, Aaron E. Lin, Robert E. Schwartz, Alexander Ploss

**Affiliations:** 1 Department of Molecular Biology, Princeton University, Princeton, New Jersey, United States of America; 2 Division of Gastroenterology and Hepatology, Department of Medicine, Weill Cornell Medicine, New York, New York, United States of America; Beijing Children’s Hospital Capital Medical University, CHINA

## Abstract

Usutu virus (USUV) is an emerging mosquito-borne flavivirus known to induce neuroinvasive disease in birds, mice, and humans in European and African countries. The mechanisms of infection and dissemination remain poorly understood. Thus, elucidating how USUV spreads in a susceptible host is crucial for identifying therapeutic targets. To investigate host defenses against USUV, we generated an infectious clone of the TC508 isolate. After characterizing its replication dynamics in cultured cells from multiple species, we investigated its pathogenesis in an array of mice with genetic perturbations. Previous studies demonstrated that whole-body deletion of type I interferon (IFN) signaling led to widespread USUV infection and fatality in mice. Here, we observed the same lethal phenotype in STAT1-deficient mice and identified hematopoietic cells specifically as central to USUV pathogenesis in a mammalian host. Deletion of STAT1 in all hematopoietic subsets, but not hepatocytes, neurons, macrophages or conventional dendritic cells, was sufficient for systemic viral dissemination and ultimate fatality. Conversely, mice lacking functional B, T, and natural killer (NK) cells but with intact myeloid cells were resistant to USUV. Our findings provide new insights into the tissue-specific barriers that regulate USUV infection and underscore the importance of innate immunity in host defense for this important emerging flavivirus.

## Author summary

Mosquito-borne viruses are continual threats to human and animal health globally. With climate change, the geographic distribution of mosquitoes is projected to expand, leading to increased burden of their accompanying viruses. The flavivirus genus consists of well-known threats including yellow fever virus, Zika virus, dengue viruses, West Nile virus, and Japanese encephalitis virus as well as closely related, but understudied species, such as Usutu virus. Although most Usutu virus infections in humans are asymptomatic, symptoms of neuroinvasive disease in individuals have been recorded. Further, this virus is highly lethal in avian species and has caused massive die-offs of Eurasian blackbirds. Here, we investigated Usutu virus infection in various cell lines and mouse strains to gain insight into how this virus establishes infection in a susceptible host. Cell lines derived from humans, mice, chicken, and mosquitoes were all susceptible to Usutu virus. Immunocompetent mice were resistant to infection, but mice with disrupted innate immune signaling succumbed to lethal infection. Notably, intact innate immune signaling was essential in hematopoietic cells, but dispensable in liver and neuronal cells. Additionally, B, T, and natural killer cells were not necessary to restrict Usutu virus infection.

## Introduction

Usutu virus (USUV) is an emerging flavivirus, which was first isolated in 1959 in Eswatini from *Culex neavei* mosquitoes [1]. USUV belongs to the Japanese encephalitis virus (JEV) serocomplex and is closely related to West Nile Virus (WNV), St. Louis encephalitis virus (SLEV), and Murray Valley encephalitis virus (MVEV). Currently, USUV is endemic in several European and African countries and was likely introduced into Europe by infected migratory birds [2]. Similar to WNV, it is maintained in the environment in an enzootic cycle between ornithophilic mosquito vectors, most commonly *Culex pipiens*, and avian amplifying hosts [3–6]. High pathogenicity and virulence of USUV has been observed in European wild birds, particularly Eurasian blackbirds (*Turdus merula*) [7–13]. USUV has been detected in a wide range of other animal hosts including human, bat, wild boar, tree squirrel, dog, horse, rat, shrew, and multimammate mouse, which are considered to be incidental hosts [2,14–20].

While the majority of USUV infections in humans are asymptomatic and have been retrospectively discovered through seroprevalence studies, such as blood donor screening [21–26], this virus has demonstrated its role as a human pathogen with a neuroinvasive capacity. In Italy in 2009, the first cases of USUV with neuroinvasive symptoms, including meningoencephalitis, distal resting tremors, dysmetria, and headache were observed in immunocompromised patients [27,28]. Subsequent neuroinvasive USUV infections have been predominantly recorded in immunocompromised patients, including one fatality [29]; however, there have also been instances of immunocompetent individuals with neurological presentation [30,31]. Separately, an *ex vivo* study demonstrated the ability of USUV to infect all three cell types comprising the human blood brain barrier in primary brain microvascular endothelial cells, astrocytes, and pericytes [32]. Albeit at a small scale compared to established flavivirus threats, USUV has clearly demonstrated a pathogenesis in humans. Understanding the mechanisms by which this virus causes disease in mammalian hosts is crucial for future research regarding vaccines or therapeutics and for preparedness efforts in the event that strains with increased human virulence emerge.

Utilizing small animal models, such as mice, is an invaluable method for studying viral pathogenesis. Adult immunocompetent mice are often resistant to flavivirus infection, including USUV; therefore, it is common to use immunocompromised mouse strains, such as mice with disrupted interferon signaling. Established mouse models to study USUV pathogenesis include suckling mice and interferon (IFN) alpha/beta receptor 1 deficient (*Ifnar1*^-/-^) mice. One group observed neuroinvasive USUV infection in 1-week-old outbred NMRI suckling mice marked by neuronal apoptosis and demyelination [33]. Another group found that Swiss suckling mice (4–7 days old) succumbed to lethal USUV infection and detected viral RNA in brain tissues, yet adult mice were resistant [34]. *Ifnar1*^-/-^ mice also serve as a lethal infection model that can be used to study USUV neuropathogenesis [35,36] or to compare the virulence of different USUV strains [37–39].

The objective of the present study was to determine the role of innate immune signaling in controlling USUV spread *in vivo*. To accomplish this, we generated an infectious clone of the Vienna 2001 isolate, USUV TC508. We confirmed its infectivity in cell lines from several species, then proceeded with *in vivo* experiments. We determined that a variety of inoculation doses and injection routes caused uniform lethality in *Ifnar1*^-/-^ and *Stat1*^-/-^ mice. Notably, we found that mice lacking functional B, T, and natural killer (NK) cells were similarly resistant to infection compared to wild-type (WT) mice, which indicated the importance of innate immune responses rather than adaptive for controlling and overcoming USUV infection. Further, by generating and infecting tissue-specific STAT1 knockout (KO) mice, we discovered that functional IFN-mediated defenses in the hematopoietic compartment were essential for survival, yet dispensable in hepatocytes and neuronal cells. We also demonstrated that IFN-β treatment before or after USUV infection partially protected mice from lethality. Ultimately, we established a new mouse model to study USUV pathogenesis and narrowed down the key cellular reservoirs of USUV to hematopoietic cells that are not classical dendritic cells or macrophages.

## Methods

### Ethics statement

All animal experiments described in this study were performed in accordance with protocols that were reviewed and approved by the Institutional Animal Care and Use and Committee (IACUC) of Princeton University (number 3063). All mice were maintained in facilities accredited by the Association for the Assessment and Accreditation of Laboratory Animal Care (AAALAC). All work with infectious USUV was reviewed and approved by the Institutional Biosafety Committee of Princeton University (protocol registration number 1145).

### Cell lines and antibodies

Hepa1-6 (CRL-1830), L929 (CCL-1), H2.35 (CRL-1995), AML12 (CRL-2254), NIH 3T3 (CRL-1658), and LMH (CRL-2117) cell lines were obtained from the American Type Culture Collection (ATCC, Manassas, VA). Huh7 (RRID:CVCL_0336), Huh7.5 (RRID:CVCL_7927) and STO5 (CRL-1503) cells were kindly provided by Dr. Charles Rice at The Rockefeller University. Hep-56.1D (RRID:CVCL_5769) cells were purchased from CLS Cell Line Service GmbH (Eppelheim, Germany). Aag2 cells were a kind gift from Dr. Raul Andino at the University of California, San Francisco. LMH cells were maintained in Waymouth’s medium (Thermo Fisher Scientific, Waltham, MA) supplemented with 10% (vol/vol) fetal bovine serum (FBS, Phoenix Scientific, Laguna Niguel, CA) and 1% (vol/vol) Penicillin-Streptomycin (P/S, Thermo Fisher Scientific, Waltham, MA). Aag2 cells were maintained in Leibovitz’s L-15 medium (Thermo Fisher Scientific, Waltham, MA) supplemented with 20% (vol/vol) FBS and 1% (vol/vol) P/S. All other cell lines were maintained in Dulbecco’s Modified Eagle Medium (DMEM) with high glucose and pyruvate (Thermo Fisher Scientific, Waltham, MA), which was supplemented with 10% (vol/vol) FBS and 1% (vol/vol) P/S. LMH cells were grown on plates coated with 0.1% (vol/vol) gelatin (MilliporeSigma, Burlington, MA) in Dulbecco’s phosphate-buffered saline (DPBS, Thermo Fisher Scientific, Waltham, MA) and incubated at 37°C with 5% CO_2_. Aag2 cells were incubated at 28°C without additional CO_2_. All other cell lines were incubated at 37°C with 5% CO_2_. All antibodies used in this study are listed below in Table 1.

**Table 1:** Primary and secondary antibodies used in this study for virus titration, flow cytometry, or histology staining.

Antibody and stock concentration	Manufacturer	Identifier	Dilution	Final concentration
Anti-mouse Flavivirus group antigen (D1-4G2-4–15 (4G2)) (1 mg/mL)	Bio-Techne	NBP2–52709	1:100 (flow) 1:1000 (FFU)	10 µg/mL (flow) 1 µg/mL (FFU)
Goat anti-mouse IgG (H + L) Cross-Adsorbed Secondary Antibody, HRP (1 mg/mL)	Thermo Fisher Scientific	G-21040	1:500	2 µg/mL
Goat anti-mouse IgG (H + L) Highly Cross-Adsorbed Secondary Antibody, Alexa Fluor 647 (1 mg/mL)	Thermo Fisher Scientific	A-21236	1:250	4 µg/mL
Anti-rabbit Japanese encephalitis virus NS3 (1 mg/mL)	GeneTex	GTX125868	1:100	10 µg/mL
Anti-mouse CD45 monoclonal antibody (HI30) (0.5 mg/mL)	Thermo Fisher Scientific	14-0459-82	1:100	5 µg/mL
CD68 monoclonal antibody (KP1) (0.2 mg/mL)	Thermo Fisher Scientific	MA5–13324	1:50	4 µg/mL
Donkey anti-rabbit IgG (H + L) Highly Cross-Adsorbed Secondary Antibody, Alexa Fluor 488 (2 mg/mL)	Thermo Fisher Scientific	A-21206	1:400	5 µg/mL
Donkey anti-mouse IgG (H + L) Highly Cross-Adsorbed Secondary Antibody, Alexa Fluor 546 (2 mg/mL)	Thermo Fisher Scientific	A10036	1:400	5 µg/mL

### USUV reconstitution and preparation

The clinical isolate USUV TC508 was originally collected from an infected blackbird in Vienna, Austria in 2001 (GenBank: AY453411.1) and was kindly provided by Dr. Kenneth Plante at the World Reference Center for Emerging Viruses and Arboviruses (WRCEVA) at the University of Texas Medical Branch (UTMB) for this study. The lyophilized virus was resuspended in 1 mL of chilled DPBS and propagated once in Huh7.5 cells. Cell culture supernatant was collected at 48, 72, and 96 hours post-infection (hpi) and stored at 4°C between timepoints. Cellular debris was removed from the pooled supernatant using a 0.45-μm syringe filter. The filtered supernatant was subsequently concentrated using the Amicon Ultra-15 Centrifugal Filter Unit (MilliporeSigma, Burlington, MA) and aliquoted in cryovials for storage at -80°C.

### Viral genome sequencing

We performed viral genome sequencing using an RNase H-based rRNA depletion method as described previously [40]. Following RNA extraction from filtered cell culture supernatant, we depleted rRNA with complementary DNA probes and RNase H and purified remaining RNA. To prepare sequencing libraries, we synthesized first-strand cDNA using random primers, synthesized second-strand cDNA using the Gubler-Hoffman method, tagmented the cDNA using the Nextera XT DNA Library Preparation Kit (Illumina, San Diego CA), and amplified cDNA fragments with indexing primers for 18 cycles of PCR. We pooled and sequenced libraries on MiSeq Micro v2 flowcells (Illumina, San Diego CA) with 2 x 151 bp reads. Following sequencing, we demultiplexed reads with Picard v2.25.6 from within viral-core v2.3.1. We performed reference-guided *de novo* viral genome assembly using viral-pipelines v2.1.33.14 [41,42]. Briefly, we removed human and bacterial contaminant reads, deduplicated the remaining reads, and filtered to genus-level (flavivirus) reads. We used these reads to assemble contigs, orient them relative to a reference genome, create a consensus genome assembly, and then re-map reads to the assembled genome to call intrahost single-nucleotide variants (iSNVs). The sequencing data was visualized using SnapGene ver. 8.0.1 (GSL Biotech LLC, Boston, MA). The sequencing data is available on GenBank at the BioProject accession PRJNA1263528.

### Generation of an USUV infectious clone by circular polymerase extension reaction (CPER)

Circular polymerase extension cloning (CPEC) was originally established for assembling complex gene libraries [43]. In 2013, this method was first adapted for the generation of infectious flavivirus cDNAs and has since been utilized by several groups including our own [44–49]. Based on the empirically determined genomic sequence for USUV TC508, seven gBlock gene fragments were obtained (Integrated DNA Technologies (IDT), Newark, NJ) and cloned into pCR-Blunt II-TOPO vectors (Thermo Fisher Scientific, Waltham, MA). An additional fragment encoding the polyA signal, cytomegalovirus (CMV) promoter, and hepatitis delta virus ribozyme (HDVr) site was cloned into an eighth pCR-Blunt II-TOPO vector. The constructed plasmids were verified by Sanger sequencing (Eton Bioscience, San Diego, CA). In preparation for CPER, the eight fragments were generated by PCR using the Q5 High-Fidelity DNA Polymerase (New England BioLabs, Ipswich, MA) and primer pairs that have complementary ends listed in Table 2. Molar concentrations of each resulting fragment were calculated using the NEBioCalculator dsDNA Mass to Moles Converter (New England BioLabs, Ipswich, MA). To generate circular cDNA by CPER, the eight fragments were combined in equimolar concentrations (0.1 pmol each) and ligated using the PrimeSTAR GXL DNA polymerase (Takara Bio USA, San Jose, CA). The following PCR cycling was used: 98°C for 2 min, followed by 20 cycles of 10 s at 98°C, 15 s at 55°C, 12 min at 68°C, and a final extension step at 68°C for 12 min. The CPER product was stored at -20°C. For transfection, Huh7.5 cells were seeded at a density of 1e6 cells/well in a 6-well plate format. The CPER product was divided and transfected into two wells of the 6-well plate using the X-tremeGENE HP DNA Transfection Reagent (MilliporeSigma, Burlington, MA). After 24 h, the contents of the two transfected wells were combined and expanded in a 100 mm plate. Additionally, two wells of non-transfected Huh7.5 cells were similarly combined as a mock control. Cells were monitored daily for cytopathic effect (CPE). Each day post-transfection, the supernatant was aspirated and replaced with warmed DMEM containing high glucose and pyruvate + 2% (vol/vol) FBS + 0.2% (vol/vol) P/S. Beginning on the first day of observed CPE, supernatant was collected and stored at 4°C. For the following two days or until 80% of cells were lifted from the plate surface, supernatant was collected and pooled. On the final day, the pooled supernatant was centrifuged at 3,000 rpm at 4°C for 10 min. The supernatant was then passed through a 0.45-μm filter to remove cellular debris. The virus was kept on ice, aliquoted into cryovials, and stored at -80°C until future use.

**Table 2:** PCR primers for amplification of USUV fragments for infectious clone generation via circular polymerase extension reaction (CPER).

Fragment #	Fragment components	Length (bp)	Primer names	F primer sequence (5’ to 3’)	R primer sequence (5’ to 3’)
1	5’ UTR, C, prM, partial E	2019	F: PU-O-8811 R: PU-O-8812	AGATGTTGGCCTGTGTGAGCTC	GAGATCGGAAAGTGATGCCAC
2	Partial E, NS1, partial NS2A	1944	F: PU-O-8813 R: PU-O-9147	GTGGCATCACTTTCCGATCTC	CATCCAAGCTGTGGCAGTGG
3	Partial NS2A, NS2B, partial NS3	2049	F: PU-O-9148 R: PU-O-8816	CCACTGCCACAGCTTGGATG	CAGCACTAGCACTAGTGATTG
4	Partial NS3	726	F: PU-O-8817 R: PU-O-8818	CAATCACTAGTGCTAGTGCTG	CGAGGAGCAAGAAAACTCCTG
5	NS4A, 2K, NS4B	1120	F: PU-O-8819 R: PU-O-9149	CAGGAGTTTTCTTGCTCCTCG	GCCTCTTTCCTGTACTTCAG
6	Partial NS5	1825	F: PU-O-9150 R: PU-O-9151	CTGAAGTACAGGAAAGAGGC	CAATCACTCCTTCAGCTTCC
7	Partial NS5, 3’ UTR	1506	F: PU-O-9152 R: PU-O-8824	GGAAGCTGAAGGAGTGATTG	AGATCCTGTGTTCTTCTCCA
8	HDVr, CMV promoter	1076	F: PU-O-8825 R: PU-O-8826	GGTGGAGAAGAACACAGGATCTGGGTCGGCATGGCATCTCCACCTC	GAGCTCACACAGGCCAACATCTCGGTTCACTAAACGAGCTCTG

### Virus titration by focus-forming unit (FFU) assay

Huh7.5 cells were seeded in a collagen-coated 48-well plate at 80% confluency. The following day, the cells were incubated with 10-fold serial dilutions of the virus stock in DPBS for 2 h at 37°C. Each dilution was tested in triplicate. Following virus adsorption, inoculum was aspirated and warmed methylcellulose overlay solution (1% (vol/vol) methylcellulose, 1% (vol/vol) DMEM, 10% (vol/vol) FBS, 1% (vol/vol) P/S + 1.85 g NaHCO_3_) was added to each well. Three days later, the overlay medium was aspirated and the cell monolayer was washed twice using unsupplemented DMEM and twice using DPBS, followed by fixation with 4% (vol/vol) paraformaldehyde (PFA, MilliporeSigma, Burlington, MA) for 15 min at room temperature. Cells were then permeabilized with 0.2% (vol/vol) Triton X-100 in DPBS for 15 min, washed with PBS, then blocked with 0.2% (vol/vol) BSA in DPBS for 30 min. Each well was stained with pan-flavivirus anti-E primary antibody clone 4G2 (Bio-Techne, Minneapolis, MN) and incubated on a rotator for 1 h at room temperature. Following two washes with DPBS, cells were stained with goat anti-mouse IgG (H + L) cross-adsorbed HRP (Thermo Fisher Scientific, Waltham, MA) secondary antibody and incubated on a rotator for 1 h. All antibody dilutions and final concentrations can be found in Table 1. After two washes with DPBS, foci were visualized using DAB Substrate Kit, Peroxidase (HRP), with Nickel, (3,3’-diaminobenzidine) (Vector Laboratories, Burlingame, CA). Deionized water was used for the final wash, then the plates were laid face down on paper towels to dry. Finally, the number of foci per well was quantified and expressed as FFU per milliliter.

### Flow cytometry

To determine the permissiveness and replication kinetics of USUV TC508 in different cell lines, cells were seeded in a 48-well plate at a density of 5e4 cells/well. Virus inoculation was performed with the indicated MOI for 2 h at 37°C. Following incubation, the inoculum was aspirated and replaced with 0.5 mL/well of DMEM containing high glucose and pyruvate + 10% (vol/vol) FBS + 1% (vol/vol) P/S. For replication kinetic characterization, cells were collected on 1–4 days post-infection (dpi). For testing the permissiveness of various murine cell lines to USUV, cells were only collected at 4 dpi. On collection days, cells were washed with DPBS, treated with 0.05% (vol/vol) trypsin in Hank’s balanced salt solution (Thermo Fisher Scientific), then quenched with supplemented cell culture medium. Following a wash with DPBS, cells were fixed in 4% (vol/vol) PFA. All samples were collected in triplicate. Uninfected wells for each cell line were also harvested and prepared for flow cytometry as mock controls. On staining days, cells were permeabilized using 0.1% (vol/vol) saponin + 1% vol/vol FBS in DPBS for 15 min at room temperature. Cells were stained with pan-flavivirus anti-E primary antibody clone 4G2 (Bio-Techne, Minneapolis, MN) and incubated for 1 h at 4°C covered in foil. Following two washes with DPBS, cells were stained with goat anti-mouse IgG (H + L) cross-adsorbed Alexa Fluor 647 (Thermo Fisher Scientific, Waltham, MA) secondary antibody. All antibody dilutions and final concentrations can be found in Table 1. Cells were then washed twice with PBS, resuspended in PBS, and transferred to flow cytometry cluster tubes. Samples were covered in foil until flow cytometry acquisition using the FACSymphony A3 with HTS Configuration (BD Biosciences, Franklin Lakes, NJ). The R-670 detector was used to determine the percentage of E-positive cells. Results were analyzed using FlowJo v10 software (BD Biosciences, Franklin Lakes, NJ).

### Mouse strains

C57BL/6 (JAX stock #000664) [50], NRG (JAX stock #007799) [51], and mice expressing Cre recombinase under the control of the CMV (CMV-Cre, JAX stock #003465) [52], albumin (Alb-Cre, JAX stock #035593) [53], Vav1 (Vav-Cre, JAX stock #008610) [54] synapsin 1 (Syn-Cre, JAX stock #003966) [55], lysozyme 2 (LysM-Cre, JAX stock #004781) [56], or integrin alpha x gene (CD11c-Cre, JAX stock #008068) [57] promoters were commercially obtained from the Jackson Laboratory (Bar Harbor, ME). Mice deficient for type I IFN receptor (*Ifnar1*^-/-^) on a C57BL/6 genetic background were kindly provided by Dr. Sergei Kotenko at Rutgers New Jersey Medical School and generated as described previously [58]. *Stat1*^fl/fl^ mice were kindly provided by Dr. Lothar Hennighausen (National Institute of Health, Bethesda, MD) [59] and backcrossed for 10 generations to the C57BL/6 background as previously described [60]. *Stat1*^-/-^, Vav-Cre/*Stat1*^fl/fl^, Alb-Cre/*Stat1*^fl/fl^, Syn-Cre/*Stat1*^fl/fl^, LysM-Cre/*Stat1*^fl/fl^, and CD11c-Cre/*Stat1*^fl/fl^ mice were generated by intercrossing *Stat1*^fl/fl^ and CMV-Cre, Vav-Cre, Alb-Cre, Syn-Cre, LysM-Cre, or CD11c-Cre mice, respectively. Vav-Cre/*Stat1*^fl/fl^, Alb-Cre/*Stat1*^fl/fl^, and Syn-Cre/*Stat1*^fl/fl^ offspring were genotyped by PCR using Bullseye Taq-DNA Polymerase (Midwest Scientific) using primer combinations to distinguish wild-type and mutant alleles (available upon request). LysM-Cre/*Stat1*^fl/fl^, and CD11c-Cre/*Stat1*^fl/fl^ mice were genotyped by the third party vendor (Transnetyx, Cordova, TN). All mice were bred in the Laboratory Animal Resource (LAR) Center of Princeton University.

### Mouse infections and monitoring experiments

Adult (6- to 14-week-old) male and female mice were infected through subcutaneous injection via nape of the neck, intravenous injection via tail vein, intramuscular via hindlimb, intraperitoneal, or intradermal via footpad with doses ranging from 10^1^–10^5^ FFU of USUV TC508 diluted in DPBS to a final volume of 200 µL. Signs of disease progression were recorded through weight measurements and clinical scoring daily. Overall appearance was assessed using a clinical scoring matrix assigned as follows: 0, posture normal, appearance with smooth, shiny fur; 1, posture hunched, appearance with ruffled fur, loss of muscle tone, loss of weight; 2, posture hunched, trembling, shaky, loss of weight; 3, posture severely hunched, disheveled appearance, significant (greater than or equal to 20%) weight loss, buildup of white residue around the eyes; 4, death. Mice with weight loss greater than or equal to 20% were euthanized by CO_2_ chamber and their deaths were recorded for the following day.

### Serum and tissue collection

Blood (ca. 200 µL) was collected through submandibular bleeding. Serum was separated from blood cells by centrifugation (3,500 rpm, 10 min, room temperature) and stored at -80°C in aliquots of 25 µL for later quantification of viremia. At the indicated endpoints, mice were anesthetized with ketamine/xylazine injection, and subject to cardiac puncture for a terminal blood collection. Spleen, liver, kidney, lung, and brain were harvested from mock or infected mice. Tissue sections were placed in 500 µL of RNA*later* stabilization solution (Thermo Fisher Scientific, Waltham, MA) and stored at -80°C for downstream molecular analysis including RNA extraction and RT-qPCR. Tissue sections were placed in 3 mL of 10% neutral buffered formalin (MilliporeSigma) for 72 h at 4°C, then washed with DPBS and stored at 4°C in 5 mL of 70% (vol/vol) ethanol for downstream histological analysis.

### RNA extraction from mouse serum and tissue

Tissue sections stored in RNAlater were thawed on ice. 10 mg of tissue was resuspended in 200 µL of MagMAX lysis/binding solution concentrate (Thermo Fisher Scientific, Waltham, MA) in a 2 mL microcentrifuge tube. A single 5-mm stainless steel bead (QIAGEN, Hilden, Germany) was placed into each tube containing tissue. Homogenization was accomplished using a TissueLyser LT (QIAGEN) set to 50 pulses per min for 2 min. After two rounds of homogenization separated by 2-min incubation on ice, the tubes were centrifuged at high speed (10,000 rpm) for 10 min at 4°C. The resulting supernatant was transferred to a 1.5-mL tube and used for viral RNA extraction. Viral RNA was isolated from 25 µL of serum or 10 mg of homogenized tissue using the MagMax mirVana Total RNA Isolation Kit (Thermo Fisher Scientific, Waltham, MA) and the KingFisher Flex machine (Thermo Fisher Scientific, Waltham, MA) according to manufacturer’s instructions. For each organ, two cuts (each 10 mg) were processed as biological replicates. For each sample, 30 µL of eluate was transferred to 0.2-mL strip tubes (USA Scientific, Ocala, FL) and subjected to DNase I treatment with Turbo DNase (Thermo Fisher Scientific, Waltham, MA). Lastly, viral RNA was purified by a 0.8x SPRI reaction using RNAClean XP beads (Beckman Coulter, Brea, CA). Purified RNA was stored in 0.2 mL strip tubes at -80°C until downstream RT-qPCR analysis.

### Reverse transcription-quantitative polymerase chain reaction (RT-qPCR)

Viral RNA was quantified using single-step RT-quantitative real-time PCR (Luna Universal One-Step RT-qPCR Kit, New England BioLabs Inc., Ipswich, MA) according to manufacturer’s protocol. To detect USUV RNA, primers PU-O-5812 (CAAAGCTGGACAGACATCCCTTAC) and PU-O-5813 (CGTAGATGTTTTCAGCCCACGT) were designed complementary to a region in USUV NS5. For each reaction 2 µL of total RNA was used as sample input. An RNA standard with a stock concentration of 9.38e8 RNA copies/µL was used. Nine 10-fold serial dilutions were prepared from the undiluted standard and were used to create a standard curve from which RNA copies in each sample were determined. RT-qPCR amplification was performed using a StepOnePlus Real Time PCR System (Thermo Fisher Scientific, Waltham, MA) with the following settings: 55°C for 10 min; 95°C for 1 min; 40 cycles of 95°C for 10 sec and 60°C for 1 min; 95°C for 10 sec; 65°C for 10 sec 95°C for 10 sec; 50°C for 5 sec.

### Mouse IFN-β treatment

Recombinant Mouse IFN-β1 (carrier-free) (BioLegend, San Diego, CA) was aliquoted upon arrival and stored at -80°C. To prepare injections, IFN-β1 was diluted in sterile DPBS and administered at a dose of 10^4^ IU/200 uL via subcutaneous route. This dose was chosen based on the methods of previous groups [61–63].

### Histological analysis

Freshly isolated mouse tissues were fixed in 4% (w/vol) paraformaldehyde for 24 h at 4°C, dehydrated, and embedded in paraffin. Tissue sectioning and paraffin processing was done by Saffron Scientific Histology Services, LLC (Illinois, USA). Sections of 5-µm thickness were obtained using a microtome. Tissue sections underwent staining with hematoxylin and eosin (H&E) using standard protocols. Stained slides were imaged using the Zeiss Axioscan 7 Microscope Slide Scanner.

### Tissue immunostaining

Unstained tissue sections were deparaffinized in xylene and rehydrated through graded alcohol to deionized water. Antigen retrieval was achieved by steaming sections in citrate buffer (pH 6.0) at 95°C for 20 min. After cooling to room temperature, non-specific binding sites were blocked using 10% donkey serum in DPBS for 1 h at room temperature. Sections were then incubated with primary antibodies, including anti-JEV-NS3 (GeneTex, Irvine CA), anti-mouse CD45 (Thermo Scientific, Waltham, MA), and anti-mouse CD68 (Thermo Scientific, Waltham, MA), overnight at 4°C. After washing in PBS, slides were incubated with donkey anti-rabbit IgG (H + L) Highly Cross-Adsorbed Secondary Antibody, Alexa Fluor 488 or Alexa Fluor 546 (Thermo Scientific, Waltham, MA) secondary antibody for 1 h at room temperature. All antibody dilutions and final concentrations used are listed in Table 1. Cell nuclei were counterstained with 4’6-diamidino-2-phenylindole (DAPI; Thermo Scientific, Waltham, MA). Immunostaining images were acquired using a confocal laser scanning microscope (Zeiss LSM 880).

### Statistical analyses

GraphPad Prism software (version 10) was used for statistical analysis. One sample t tests were used to compare quantitative data to the limit of detection (*P < 0.05, **P < 0.01, ***P < 0.001, ****P < 0.0001).

## Results

### Cell lines from several species are susceptible to USUV infection

**Fig 1:**
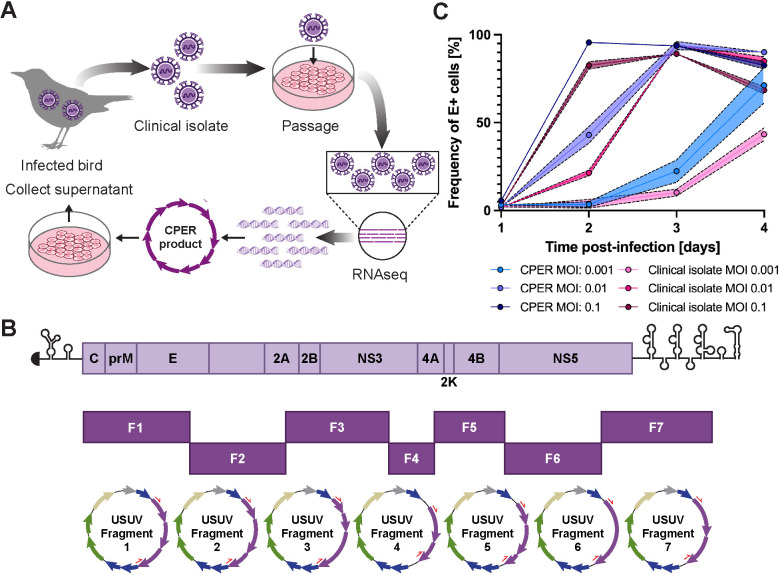
A genetically-defined infectious clone of USUV TC508 was generated using the circular polymerase extension reaction (CPER) and remains infectious in cell culture. **(A)** Schematic representation of the workflow for generating an USUV infectious clone from an avian clinical isolate. Some figure elements (bird [64], cells [65], and DNA fragments [66]) were sourced from the public domain and are listed as references. **(B)** Schematic representation of the USUV genome and its division into seven fragments used for CPER. Each fragment was inserted into pCR-Blunt II-TOPO vectors. The purple arrows represent the USUV genes. Other vector components include lacZa (blue), ccdB (blue), antibiotic resistance markers (green), ori (sand), and the lac promoter (gray). The red half arrows indicate the PCR primer binding sites. **(C)** Replication kinetics of the USUV TC508 infectious clone in human hepatoma (Huh7) cells 1–4 days post-infection (dpi) using a multiplicity of infection (MOI) of 0.001, 0.01, or 0.1. The percentage of infected cells was quantified based on envelope (E) antigen staining detected by flow cytometry. Shading indicates standard error of the mean.

For this study, we generated an infectious clone of the Vienna 2001 isolate, USUV TC508. This isolate was originally obtained from an infected blackbird, minimally passaged in cell culture, then subjected to RNA sequencing (RNAseq). The USUV TC508 genome was subdivided into 7 fragments which were then combined using a circular polymerase extension reaction (CPER) [44,45]. Upon transfection into human Huh7.5 hepatoma cells, the resulting CPER products yielded full-length viral genomic transcripts driven by a CMV promoter with a native 3’ end produced by a hepatitis delta virus ribozyme (Fig 1A and 1B). We ensured the genome sequence of our CPER-derived virus matched the clinical isolate by RNAseq. Since hepatic damage is commonly observed during flavivirus infection and has been observed in USUV-infected birds and mice, we set out to characterize the replication kinetics of our CPER-derived USUV in cell lines originating from the liver of several species [7,12,37,67]. We first tested the permissiveness of a human (Huh7) hepatoma cell line to the CPER-derived USUV versus the original clinical isolate using three multiplicities of infection (MOI). We determined the percentage of infected cells by quantification of USUV envelope (E) antigen-bearing cells by flow cytometry. At all tested MOIs, we recorded similar replication kinetics between the CPER-derived virus and the clinical isolate; however, the CPER-derived virus was consistently higher (Fig 1C). At the lowest tested MOI (0.001), the percentage of E-positive cells was 3% at 1 and 2 dpi following exposure to the CPER-derived virus and increased to 22% on 3 dpi and 71% on 4 dpi. Similarly, using the clinical isolate, 23% of cells were E-positive on 1 and 2 dpi, which progressed to 10% on 3 dpi and rose to 43% on 4 dpi. Escalating to an MOI of 0.01, 2% of cells were E-positive at 1 dpi using the CPER-derived virus, which grew to 43% by 2 dpi, peaked to 94% at 3 dpi, and decreased to 90% at 4 dpi due to cytopathic effect (CPE) and cell death. Using the clinical isolate at the same MOI, the frequency of E-positive cells increased from 2–21% at 1–2 dpi, peaked to 94% at 3 dpi, and fell to 85% at 4 dpi. At the highest tested MOI (0.1), infection progressed quickly between 1–2 dpi as we detected an increase in E-positive cells from 6 to 96% using the CPER-derived virus or 2–83% using the clinical isolate. Infection rates remained high as 94% and 83% of CPER-derived virus-infected cells were E-positive on 3–4 dpi and 89% and 68% of clinical isolate-infected cells were E-positive on 3–4 dpi. Ultimately, we found that the intermediate MOI of 0.01 provided the most steady USUV growth kinetics from 1–4 dpi. Using the higher MOI of 0.1, the vast majority of cells were infected by 2 dpi and then decreased by 4 dpi due to cell death. Using the lower MOI of 0.001, peak infection was likely not achieved; however, we did not want to extend the experiment due to cell overgrowth. Moving forward, we proceeded with using an MOI of 0.01 to test the susceptibility of other cell lines.

**Fig 2:**
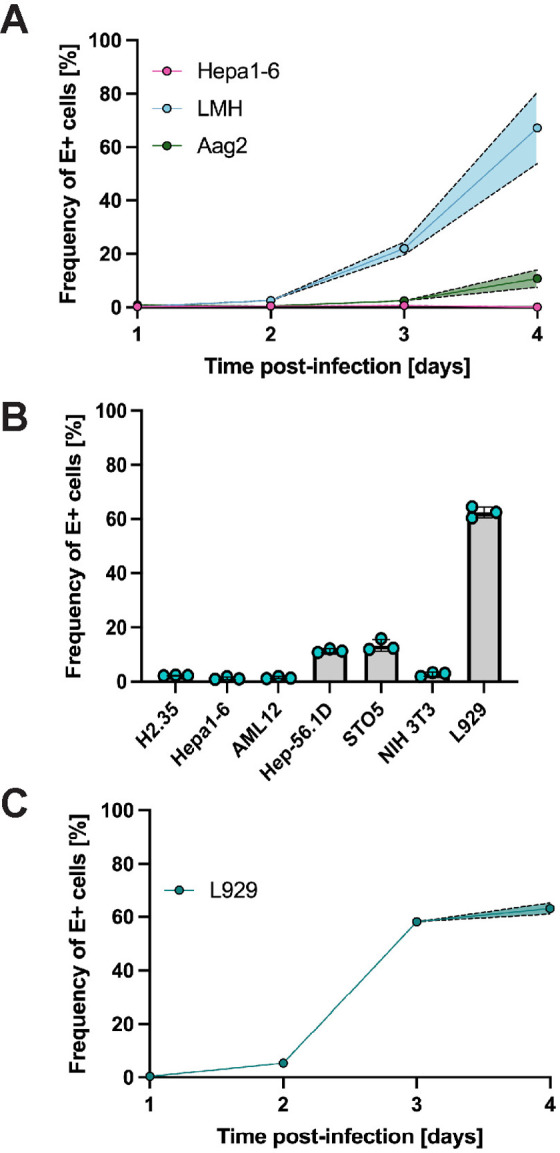
USUV TC508 has variable Infection kinetics in murine, avian, and mosquito cell lines. **(A)** Percent of E antigen-positive cells in mouse hepatoma (Hepa1-6), chicken hepatoma (LMH), and embryonic mosquito cells (Aag2) 1–4 days post-infection (dpi) using a multiplicity of infection (MOI) of 0.01. Shading indicates standard error of the mean. **(B)** Percent of E antigen-positive cells in various murine cell lines at 4 dpi using an MOI of 0.01. **(C)** Percent of E antigen-positive cells in mouse fibroblasts (L929) 1–4 dpi using an MOI of 0.01. Shading indicates standard error of the mean.

Since mice serve as a common animal model for flavivirus research and birds are the amplifying host of USUV, we next determined the replication kinetics of USUV in hepatoma cell lines from these species, which had not been previously done. Additionally, to model replication in the vector, we tested the permissiveness of the embryonic mosquito cell line Aag2, which had also not been used for USUV studies. We previously saw robust infection of Huh7 cells using MOIs 0.001, 0.01, and 0.1, but captured the best steady increase in infection using the intermediate MOI of 0.01; therefore, we chose to proceed with an MOI of 0.01. Chicken hepatoma cells (LMH) had a steady progression of infection from 0% to 3% at 1–2 dpi, 22% at 3 dpi, and 67% at 4 dpi. Notably, mouse hepatoma cells (Hepa1–6) were non-permissive to USUV infection and mosquito cells (Aag2) demonstrated low permissiveness (11% infection) to USUV by 4 dpi (Fig 2A). Before proceeding with *in vivo* experiments, we were curious if we could find a murine cell line that was permissive to USUV. Thus, we infected the murine hepatocellular carcinoma cell lines H2.35, AML12, and Hep-56.1D and the murine fibroblast cell lines STO5, NIH 3T3, and L929, then evaluated the frequencies of E antigen-bearing cells on 4 dpi (Fig 2B). We chose this timepoint since we knew a high frequency of cells should be E-positive by 4 dpi based on our replication kinetics experiment in Huh7 cells and did not want to choose a later timepoint in which cell death or overgrowth could impede results. Hep-56.1D and STO5 cells were minimally permissive, reaching 10–15% infection at 4 dpi, while H2.35, AML12, and NIH 3T3 appeared to be resistant to USUV. L929 fibroblasts supported a comparable level of USUV infection to Huh7 and LMH cells, reaching 63% at 4 dpi. Collectively, these data demonstrate that the CPER-produced virus is infectious across multiple cell lines from various species and tissues. Further, we determined the growth kinetics of USUV in L929 cells from 1–4 dpi (Fig 2C). E-positive cells were first detected at 2 dpi and their frequency was 5%. This rose to 58% on 3 dpi and 63% on 4 dpi.

### Type I interferon receptor-deficient and STAT1-deficient mice succumb to lethal USUV infection by low or high inoculation dose

To determine if our USUV infectious clone was pathogenic *in vivo*, we subcutaneously injected mice with disrupted IFN signaling (*Ifnar1*^-/-^ and *Stat1*^-/-^) with five inoculation doses ranging from 10^1^–10^5^ focus-forming units (FFU) per mouse. On a daily basis, we measured change in weight, assessed clinical signs (ruffled fur, hunched posture, loss of muscle tone, decreased mobility, trembling, buildup of white residue around the eyes), and euthanized mice that reached ≤ 80% of their initial weight measured before infection.

**Fig 3:**
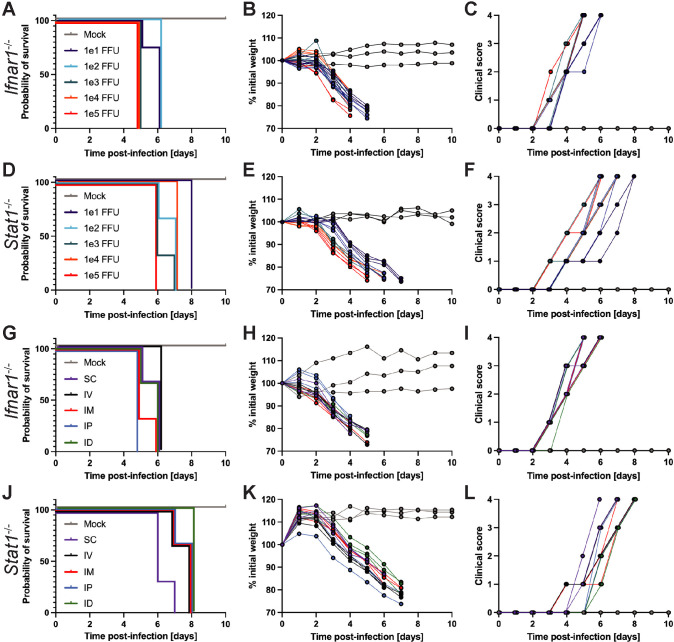
USUV infection is lethal in mice with compromised type I interferon or STAT1-mediated antiviral defenses regardless of inoculation dose or route. The inoculation doses tested ranged from 10^1^ to 10^5^ focus-forming units (FFU), all of which were administered via subcutaneous (SC) route. The inoculation routes tested were SC, intravenous (IV), intramuscular (IM), intraperitoneal (IP), and intradermal (ID) using a consistent inoculation dose of 10^3^ FFU. **(A)** Survival, (**B**) weight changes, and (**C**) clinical scoring of *Ifnar1*^-/-^ mice following inoculation with varying doses of USUV via SC injection (10^1^ FFU, n = 4; 10^2^ FFU, n = 4; 10^3^ FFU, n = 7; 10^4^ FFU, n = 3; 10^5^ FFU, n = 3). **(D)** Survival, (**E**) weight changes, and (**F**) clinical scoring of *Stat1*^-/-^ mice following inoculation with varying doses of USUV via SC injection (n = 3/group). **(G)** Survival, (**H**) weight changes, and (**I**) clinical scoring of *Ifnar1*^-/-^ mice following inoculation with 10^3^ FFU of USUV by various routes (n = 3/group). **(J)** Survival, (**K**) weight changes, and (**L**) clinical scoring of *Stat1*^-/-^ mice following inoculation with 10^3^ FFU of USUV by various routes (n = 3/group).

Regardless of the dose, all *Ifnar1*^-/-^ and *Stat1*^-/-^ mice experienced incremental weight loss, increasing clinical signs, and ultimately succumbed to lethal infection between 5–6 dpi and 6–8 dpi, respectively (Fig 3A–3F), in sharp contrast to wild-type mice of which 100% survived infection with 10^3^ FFU of USUV. In *Ifnar1*^-/-^ mice, all animals that were inoculated with 10^1^–10^2^ FFU of USUV died by 6 dpi while animals that received 10^3^–10^5^ FFU of USUV died by 5 dpi. At all doses tested, *Ifnar1*^-/-^ mice succumbed to lethal infection sooner than *Stat1*^-/-^ mice. In *Stat1*^-/-^ mice, animals that received the highest dose (10^5^ FFU) died by 6 dpi, animals injected with the lowest dose (10^1^ FFU) died by 8 dpi, and those injected with 10^2^–10^4^ FFU of USUV died within this range. Previous studies established *Ifnar1*^-/-^ mice as a lethal model for USUV infection and noted clinical signs such as lethargy, progressive weight loss, ruffled fur, and hunching [35–39]. In addition to confirming that *Ifnar1*^-/-^ mice could be used as a model for our USUV infectious clone and exhibited similar disease progression to other groups, we established *Stat1*^-/-^ mice as a new model for USUV investigation, and determined 10^3^ FFU as the optimal inoculation dose to observe uniform lethality at an early time point while also conserving the amount of virus used.

### USUV exposure by various inoculation routes causes lethal infection in *Ifnar1*^-/-^ and *Stat1*^-/-^ mice

To test if lethality was dependent on the route of inoculation, we inoculated *Ifnar1*^-/-^ and *Stat1*^-/-^ mice with 10^3^ FFU of USUV via subcutaneous (SC), intravenous (IV), intramuscular (IM), intraperitoneal (IP), or intradermal (ID) injection. Regardless of the inoculation route, 100% of *Ifnar1*^-/-^ and *Stat1*^-/-^ mice succumbed to lethal USUV infection by 6 and 8 dpi, respectively (Fig 3G and 3J). Death of *Ifnar1*^-/-^ mice inoculated via SC, IM, IP, or ID routes was first observed at 5 dpi, but 100% of IV-inoculated mice died at 6 dpi. In *Stat1*^-/-^ mice, lethality was observed most quickly following SC inoculation (6–7 dpi) while the longest time until death was observed by ID inoculation (8 dpi). *Stat1*^-/-^ mice inoculated via IV, IM, or IP all succumbed to lethality between 7–8 dpi. Following infection, incremental weight loss (Fig 3H and 3K) and additional clinical signs listed previously were recorded in *Ifnar1*^-/-^ and *Stat1*^-/-^ mice (Fig 3I and 3L). Ultimately, no specific inoculation route was associated with increased or decreased USUV pathogenesis and we used the SC injection route for all subsequent experiments.

### STAT1-mediated IFN signaling in the hematopoietic compartment is essential for restricting USUV infection

**Fig 4:**
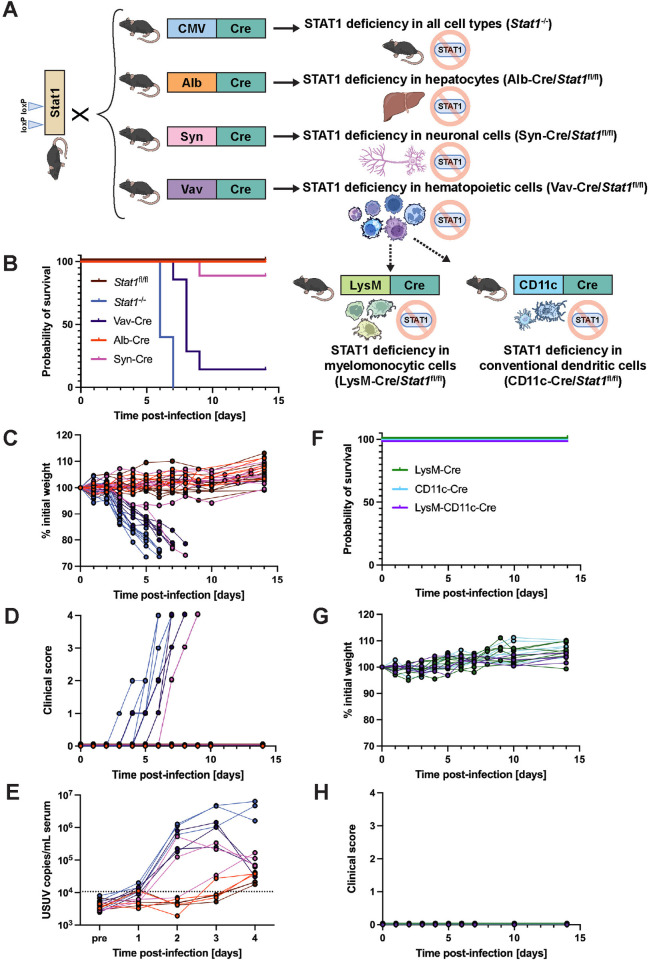
USUV infection in tissue-specific STAT1-deficient mouse strains demonstrates a necessity for functional STAT1-mediated signaling in the hematopoietic compartment. **(A)** Schematic representation of the breeding scheme used to generate whole-body, hepatocyte-specific, neuronal cell-specific, hematopoietic cell-specific, macrophage-specific, or dendritic cell-specific STAT1 KO mice. Wild-type mice harboring loxP sites within the Stat1 gene (*Stat1*^fl/fl^) were crossed with mice containing the Cre recombinase downstream of a ubiquitous (CMV) or tissue-specific (Alb, Syn, Vav, LysM, CD11c) promoter. Some figure elements (mouse [69], liver [70], neuron [71], and immune cells [72–77]) were sourced from the public domain and are listed as references. **(B)** Survival, (**C**) weight changes, and (**D**) clinical scoring of *Stat1*^fl/fl^ (n = 11), *Stat1*^-/-^ (n = 10), Vav-Cre/*Stat1*^fl/fl^ (n = 7) Alb-Cre/*Stat1*^fl/fl^ (n = 6), and Syn-Cre/*Stat1*^fl/fl^ (n = 9) mice following inoculation with 10^3^ focus-forming units (FFU) of USUV TC508 via SC injection. **(E)** Changes in viremia 1–4 days post-infection compared to a pre-bleed sample (“pre”) collected prior to infection (n = 3/mouse genotype). **(F)** Survival, (**G**) weight changes, and (**H**) clinical scoring of LysM-Cre/*Stat1*^fl/fl^ (n = 8), CD11c-Cre/*Stat1*^fl/fl^ (n = 8), and LysM-CD11c-Cre/*Stat1*^fl/fl^ (n = 4) following inoculation with 10^3^ FFU of USUV TC508 via SC injection.

To evaluate whether STAT1 was required throughout all tissues or just in certain compartments, we generated three tissue-specific STAT1 KO mice and tested their susceptibility to USUV. We chose to generate hematopoietic cell-specific STAT1 KO mice because a previous study from our group determined that these mice were hypersusceptible to infection with other orthoflaviviruses, such as yellow fever virus strain 17D, and we hypothesized that YFV and USUV may have a similar reliance on STAT1-mediated signaling in hematopoietic cells [60]. Next, we chose to generate hepatocyte-specific STAT1 KO mice since USUV has been recorded to cause hepatomegaly and liver necrosis in birds and mice [7,12,37]. Lastly, we chose to generate neuronal-cell specific STAT1 KO mice due to the demonstrated neuroinvasive potential of USUV in humans, mice, and birds [30,33,36,67]. To generate these mice, we crossed mice that harbored the Cre recombinase gene downstream of a tissue-specific promoter (Vav-Cre for hematopoietic cell expression [68], Alb-Cre for hepatocytes [53], Syn-Cre for neuronal cells [55]) to mice with loxP sites flanking exons 1 and 3 of the *Stat1* gene (*Stat1*^fl/fl^) [59] (Fig 4A). We then compared the USUV susceptibility of these mouse strains to wild-type mice (*Stat1*^fl/fl^) or full body STAT1 KO mice (*Stat1*^-/-^), which exhibited 100% survival or 100% lethality, respectively. While Alb-Cre/*Stat1*^fl/fl^ and Syn-Cre/*Stat1*^fl/fl^ mice had high survival rates of 100% and 89%, respectively, only 17% of Vav-Cre/*Stat1*^fl/fl^ mice survived after 9 dpi (Fig 4B). In the Vav-Cre/*Stat1*^fl/fl^ mice and the singular Syn-Cre/*Stat1*^fl/fl^ mouse that succumbed to lethal USUV infection, we observed incremental weight loss and the development of the same clinical symptoms as recorded in *Stat1*^-/-^ mice, albeit at a slightly delayed timeline (Fig 4C and 4D). While *Stat1*^-/-^ mice died between 6–7 dpi, lethality in Vav-Cre/*Stat1*^fl/fl^ mice occurred between 7–9 dpi and occurred at 9 dpi for the singular Syn-Cre/*Stat1*^fl/fl^ mouse. Further, we longitudinally evaluated viremia in all five mouse lines and detected high levels of USUV RNA in the serum from mice that succumbed to lethal USUV infection (Fig 4E). In Syn-Cre/*Stat1*^fl/fl^, Vav-Cre/*Stat1*^fl/fl^, and *Stat1*^-/-^ mice, there were 38-fold, 35-fold, and 68-fold increases in USUV RNA detected in serum between 1 and 2 dpi. In contrast, viral RNA levels in serum from *Stat1*^fl/fl^ and Alb-Cre/*Stat1*^fl/fl^ mice remained below the limit of detection. While viremia in *Stat1*^-/-^ mice remained high 3–4 dpi, viremia in Vav-Cre/*Stat1*^fl/fl^ peaked at 3 dpi and then decreased prior to death of the animals. Compared to these two strains, viremia in Syn-Cre/*Stat1*^fl/fl^ mice was overall lower, but still notable compared to the low levels observed in Alb-Cre/*Stat1*^fl/fl^ and *Stat1*^fl/fl^ mice. Ultimately, these data suggest that IFN signaling in hematopoietic cells is critical for protection against lethal USUV infection.

It was previously shown that mice with a targeted disruption of the IFN alpha/beta receptor in macrophages and dendritic cells (DCs) were hypersusceptible to certain DENV strains [78]. To probe whether STAT1 signaling is required for USUV clearance in these particular hematopoietic cell subsets, we crossed *Stat1*^fl/fl^ mice with mice expressing the Cre recombinase specifically in monocytes, mature macrophages, and granulocytes (LysM-Cre) [56], conventional DCs (CD11c-Cre) [57], or both (LysM-CD11c-Cre). All three mouse strains demonstrated 100% survival, maintained healthy weights, and did not develop any clinical signs throughout daily monitoring for 14 dpi (Fig 4F–4H). Collectively, these data demonstrate that STAT1-dependent signaling in monocytes, macrophages, granulocytes and conventional DCs is not required for controlling USUV infection in mice.

### USUV has broad tissue tropism and causes pathological lesions in STAT1-deficient mice

**Fig 5:**
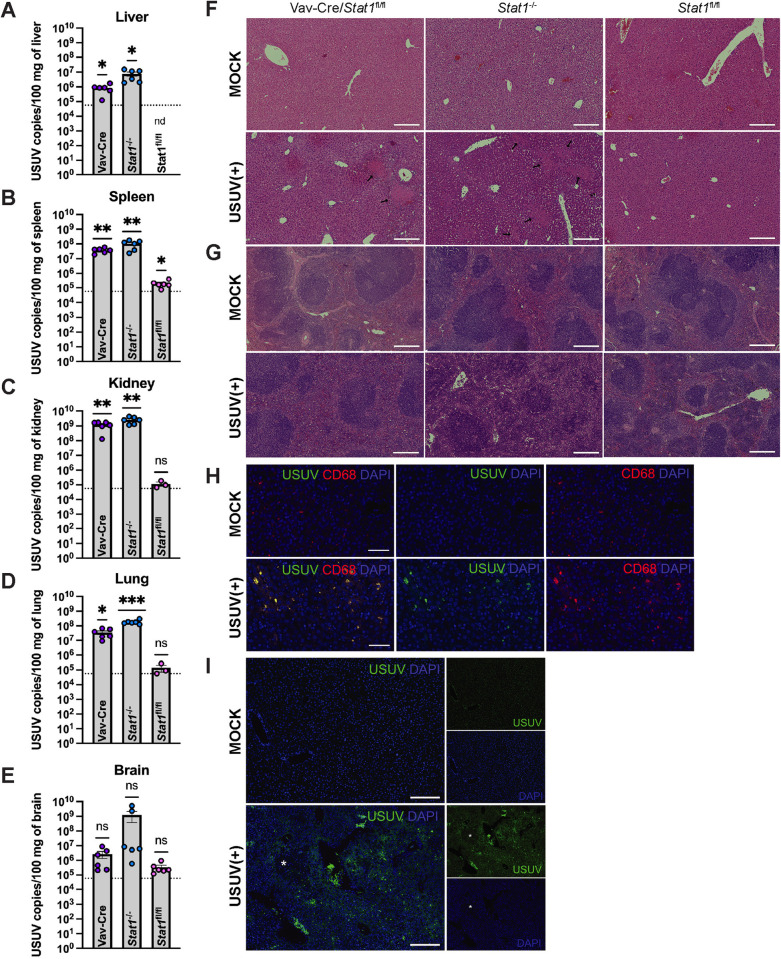
USUV replicates in STAT1-sufficient peripheral tissues leading to spleen and liver pathology. Vav-Cre/*Stat1*^fl/fl^, *Stat1*^-/-^, and *Stat1*^fl/fl^ mice (n = 3/group) were infected with 10^3^ focus-forming units (FFU) of USUV via subcutaneous (SC) injection and euthanized at 4 days post-infection (dpi). Tissues were collected and subjected to molecular and histological analysis. For each mouse genotype, one representative image was chosen. USUV RNA was quantified in the (A) liver, (B) spleen, (C) kidney, (D) lung, and (E) brain of Vav-Cre/*Stat1*^fl/fl^, *Stat1*^-/-^, and *Stat1*^fl/fl^ mice by qPCR. Significant levels of USUV RNA were detected in the liver, spleen, kidney, and lung of Vav-Cre/*Stat1*^fl/fl^ and *Stat1*^-/-^ mice. In some samples collected from *Stat1*^fl/fl^ mice (6 out of 6 liver samples, 5 out of 6 spleen samples, and 3 out of 6 kidney samples), the amount of USUV RNA was below the limit of detection and was not plotted (nd indicates “not detected”). For each organ harvested from infected mice, two cuts were processed and used as separate sample inputs for qPCR. The dotted line indicates the limit of detection. Statistical analysis was performed using one sample t tests comparing USUV RNA detected in samples to the limit of detection. **(F)** H&E staining of liver tissue collected from mock or USUV-infected mice with a 200 µm scale bar. Liver necrosis was observed in Vav-Cre/*Stat1*^fl/fl^ and *Stat1*^-/-^ mice and is indicated by black arrows. **(G)** H&E staining of spleen tissue collected from mock or USUV-infected mice with a 200 µm scale bar. Decreased red pulp due to massive immune cell infiltration was observed in Vav-Cre/*Stat1*^fl/fl^ and *Stat1*^-/-^ mice. H&E staining of kidney, lung, and brain tissues can be found in S1 Fig. **(H)** Immunostaining of liver tissue harvested from a *Stat1*^-/-^ mouse using the histiocyte marker CD68, anti-JEV NS3 to mark USUV infection, and DAPI with a 50 µm scale bar. **(I)** Liver tissue harvested from a *Stat1*^-/-^ mice and stained with anti-JEV NS3 and DAPI with a 200 µm scale bar. The white asterisk indicates an inflammatory lesion.

Due to the understudied nature of this virus, limited characterization of USUV replication, dissemination, and pathology in peripheral tissues has been accomplished in mouse models. Here, we detected high levels of USUV RNA in liver, spleen, kidney, lung, and brain from *Stat1*^-/-^ and Vav-Cre/*Stat1*^fl/fl^ mice, but not *Stat1*^fl/fl^, at 4 dpi (Fig 5A–5E). Amplification of primer dimers (based on melt curve analysis) limited our ability to quantify USUV in samples with fewer than 93.8 copies/uL of USUV RNA, including our mock tissue samples. Nevertheless, tissues with a high viral burden were still clearly identified and demonstrated a statistically significant increase in USUV RNA compared to the limit of detection. Correspondingly, histological analysis of tissues from USUV-infected mice revealed extensive necrosis and significant immune infiltration in highly vascularized organs, including the liver, spleen, and lungs, compared to mock mice. These pathological features were most pronounced in the liver and spleen of Vav-Cre/*Stat1*^fl/fl^ and *Stat1*^-/-^ mice (Fig 5F and 5G). Kidney, lung, and brain sections from Vav-Cre/*Stat1*^fl/fl^ and *Stat1*^-/-^ mice as well as all tissues from Alb-Cre/*Stat1*^fl/fl^ and Syn-Cre/*Stat1*^fl/fl^ mice can be found in the supporting information (S1 Fig). To confirm the presence of USUV in tissues, we detected USUV NS3 protein in liver tissue harvested from infected *Stat1*^-/-^ mice using an anti-JEV NS3 antibody (Fig 5H and 5I). Further, immunostaining of tissues from *Stat1*^-/-^ mice confirmed that the immune cells infiltrating the necrotic regions of the liver expressed CD68, which is a marker of histiocyte-like immune cells (Fig 5H). Counterstaining with the pan-immune cell marker CD45 can be found in the supporting information (S2 Fig). These findings demonstrate that USUV can establish infection and circulate systemically in mice with global or hematopoietic cell-specific deletion of STAT1. Further, in these mice, USUV is able to invade and cause pathology in STAT1-sufficient tissues.

### B, T and natural killer cells are dispensable for USUV clearance in mice

**Fig 6:**
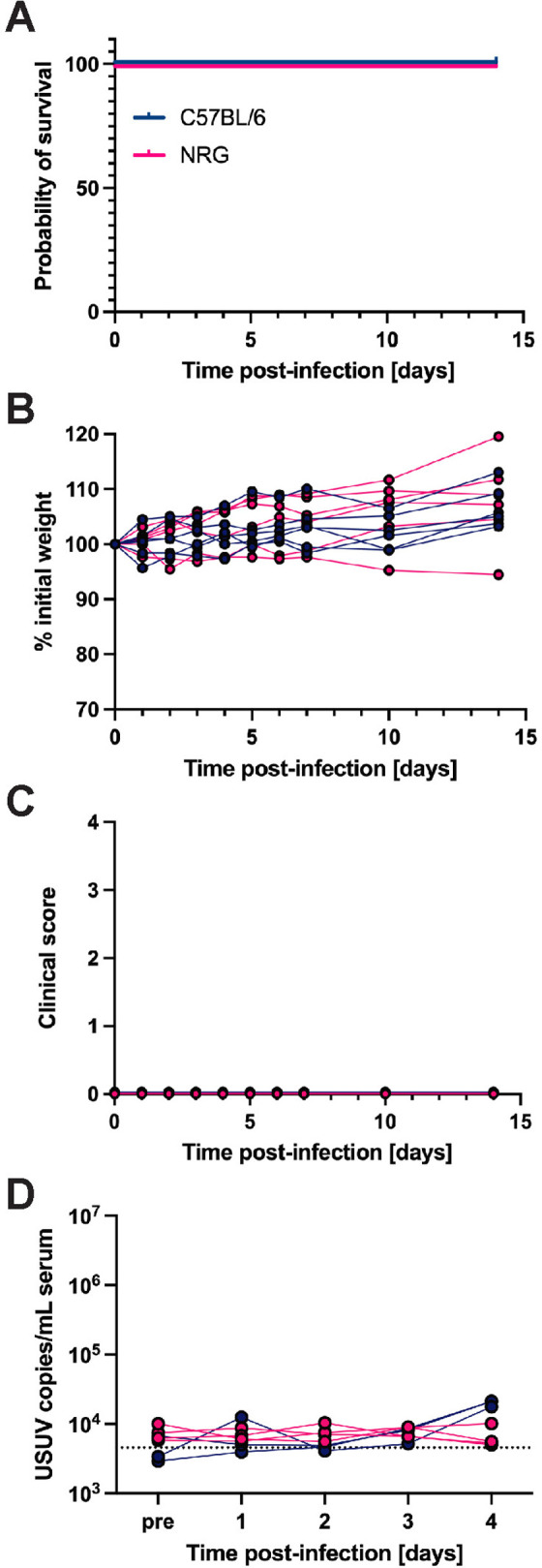
B, T, and natural killer cells are dispensable for restricting USUV infection. **(A)** Survival, (**B**) weight changes, and (**C**) clinical scoring of NRG (n = 6) and wild-type C57BL/6 (n = 6) mice following inoculation with 10^3^ focus-forming units (FFU) of USUV TC508 via subcutaneous (SC) injection. **(D)** Changes in viremia 1–4 days post-infection (dpi) compared to a pre-bleed sample (“pre”) collected prior to infection in NRG (n = 4) and C57BL/6 (n = 3) mice.

With the knowledge that STAT1-mediated IFN signaling in hematopoietic cells is essential for defense against lethal USUV infection, we questioned whether lymphocyte subsets were required. We challenged highly immunodeficient NOD-*Rag1*^*null*^*-IL2rg*^*null*^ (NRG) mice, which lack functional B, T, and natural killer (NK) cells [79]. These mice demonstrated 100% survival and the absence of weight loss and other clinical signs throughout 14 days of post-infection monitoring (Fig 6A–6C). Further, we did not detect viral RNA in serum collected from C57BL/6 nor NRG mice 1–4 dpi. NRG resistance to USUV infection indicated a nonessential role of adaptive immunity and as well as NK cell-driven innate responses in USUV clearance.

### IFN-β treatment following USUV exposure partially protects Vav-Cre/*Stat1*^fl/fl^ mice from lethality

**Fig 7:**
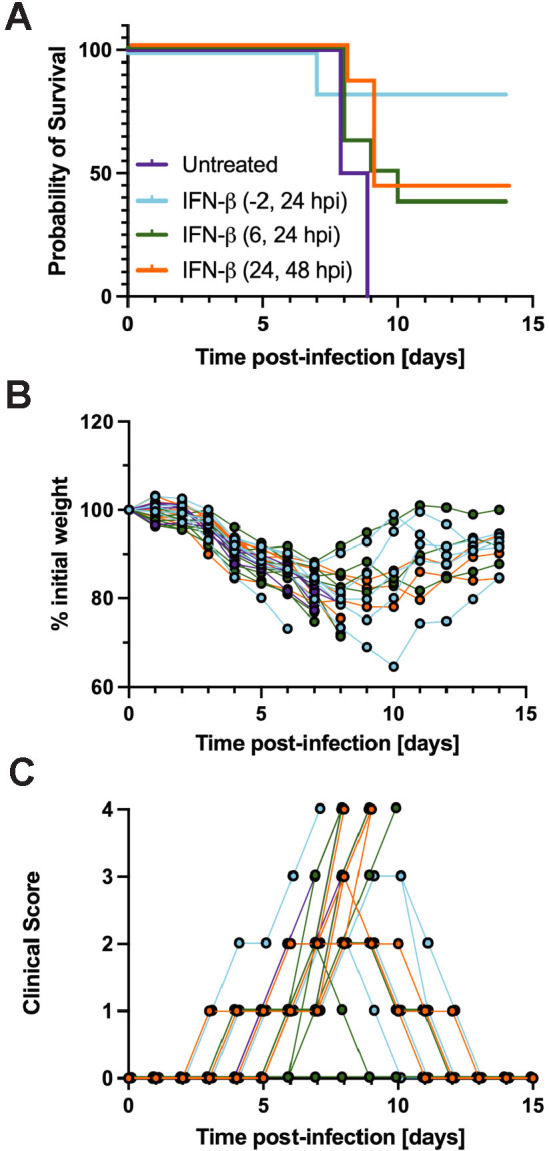
Administration of IFN-beta (IFN-β) partially protects Vav-Cre/*Stat1*^fl/fl^ mice from lethal USUV infection. **(A)** Survival, (**B**) weight changes, and (**C**) clinical scoring of Vav-Cre/*Stat1*^fl/fl^ mice that received 10^4^ IU IFN-β via SC injection at two timepoints before or after infection with 10^3^ FFU of USUV TC508. The pre-treatment group (n = 6) received IFN-β injections at 2 hours prior to USUV infection and 24 hours post-infection (hpi). One post-treatment group received IFN-β injections 6 and 24 hpi (n = 8) and the second post-treatment group received IFN-β injections at 24 and 48 hpi (n = 7).

Vav-Cre/*Stat1*^fl/fl^ mice are highly susceptible to lethal USUV infection, which may be attributable to disrupted STAT1-mediated IFN signaling in their hematopoietic cells. Thus, we investigated if administration of mouse IFN-β before or after USUV infection could provide protection against lethality. The pre-treatment group received one dose of IFN-β at 2 hours prior to USUV infection and another dose at 24 hpi. The other two treatment groups received both doses of IFN-β at two timepoints following infection: 6 and 24 hpi or 24 and 48 hpi. The untreated group did not receive any cytokine. Pre-treatment with IFN-β proved to be more effective than post-infection treatment. In the pre-treatment group (-2, 24 hpi), 5 out of 6 mice survived following USUV infection, while 4 out of 4 untreated mice succumbed to lethality by 9 dpi. In the early post-infection group (6, 24 hpi), 3 out of 8 mice survived while the others died between 8–10 dpi. In the later post-infection group (24, 48 hpi), 3 out of 7 mice survived and the rest died between 8–9 dpi. (Fig 7A). Notably, the IFN-β-treated mice that survived USUV infection had all manifested clinical signs including ruffled fur and weight loss to a similar degree as the non-surviving mice in their groups; however, they ultimately regained lost body weight and recovered (Fig 7B and 7C). Collectively, these data further affirm that early interferon signaling is critical for controlling USUV infection *in vivo*.

## Discussion

Due to the continued geographical expansion of arthropod vectors, we must further monitor and investigate both well-known and understudied flaviviruses to develop prophylactic vaccines and therapeutic options beyond supportive care [80]. To this end, we generated an infectious clone of USUV isolate TC508 and demonstrated its pathogenicity both *in vitro* and *in vivo*. Similar to established flavivirus threats, such as DENV, YFV, ZIKV, and WNV, that cause more than 400 million infections annually and have been more extensively characterized [81], USUV was highly lethal in mice bearing deficiency in type I IFN signaling. This pathway was specifically essential in hematopoietic cells in defense against USUV. Our work here is one of the first characterizations of USUV pathogenesis using adult mouse models and should be an invaluable resource for further molecular characterizations of USUV and potential medical countermeasures.

USUV has the remarkable ability to replicate in wildly divergent species—mosquito, birds, and mammals—therefore, we tested its ability to replicate in cultured cells from these species. The first study of USUV *in vitro* tested the susceptibility of cell lines from multiple species including human, rat, monkey, bovine, equine, porcine, avian, canine, feline, rabbit, hamster, and turtle. Infection with pronounced cytopathic effect (CPE) was observed in African green monkey (Vero), porcine (PK-15), and goose embryonic fibroblasts. Noncytopathic infection with positive detection of viral antigen by IHC staining occurred in all other cell lines except for chicken embryonic fibroblasts, which were not susceptible to infection [82]. Extensive summaries of *in vitro* models used to study USUV have been nicely summarized by previous groups [39,83]. In the present study, we determined that USUV successfully infected and replicated in human, bird, mosquito, and mouse lines; however, the degree of permissiveness varied widely between mouse cell lines. Overall, murine fibroblasts (STO5, NIH 3T3, L929) were more permissive compared to hepatocyte or hepatoma cell lines (Hepa1–6, H2.35, AML12, Hep-56.1D). Further, fibroblasts isolated from an adult mouse (L929) were more permissive than fibroblasts isolated from embryos (STO5, NIH 3T3). Our work establishes several new cell lines as feasible models for propagating and studying USUV infection including Huh7, LMH, and L929. Further, we have determined other cell lines to be lowly permissive to USUV such as Hepa1–6, H2.35, AML12, and NIH 3T3, which may lack necessary host factors or encode restriction factors. Future work could explore the permissiveness of human and murine neuronal cell lines as well as primary cells isolated from the blood brain barrier to better characterize the neurotropic capacity of this virus *in vitro* and *ex vivo*. Some studies have successfully infected primary cell types isolated from the human brain [32,84], but analogous studies have yet to be performed in similar cell types isolated from mice.

In devising medical countermeasures against flaviviruses, new animal models and an improved understanding of USUV pathogenesis will be required. In this study, we established *Stat1*^-/-^ and Vav-Cre/*Stat1*^fl/fl^ mice as new models for USUV investigation and confirmed that they exhibited similar disease progression to *Ifnar1*^-/-^ mice. Regardless of inoculation dose or route, USUV infection in *Stat1*^-/-^ mice was uniformly lethal by 8 dpi. Using our optimized conditions of 10^3^ FFU via SC injection, 83% of Vav-Cre/*Stat1*^fl/fl^ mice succumbed to lethal USUV infection by 9 dpi. Molecularly, lethal infection was associated with USUV dissemination to a wide range of tissues—liver, spleen, lung, kidney, and brain—based on RT-qPCR assays, as well as necrosis and significant immune cell infiltration in the liver and spleen. Of note, we observed the same outcomes in male and female mice; therefore, there were no sex differences for any genotypes used in this study. As a proof-of-concept, treating USUV-challenged mice with exogenous IFN-β led to disease recovery in some cases, particularly with pre-infection treatment. Together, our study establishes inoculation parameters, as well as expected baseline pathology, for lethal USUV infection in IFN-deficient mouse models.

While multiple groups have used *Ifnar1*^-/-^ mice to study USUV, the specific parameters by which IFN signaling protects against lethal USUV infection are not well-defined. Our data collectively suggest an important role of IFN signaling in non-macrophage, non-conventional dendritic cell subsets of the myeloid lineage during very early infection. First, we found that STAT1-mediated IFN signaling was essential in hematopoietic cells, using cell-specific deletion of STAT1 in Vav-expressing cells. Although STAT1 was only disrupted in the hematopoietic system, USUV was able to gain entry to, and replicate efficiently, in STAT1-sufficient peripheral organs. Second, we determined that B, T, and NK cells were not required for protection against USUV, as NRG mice were resistant to infection. Third, we redirected our focus towards myeloid cells and determined that deletion of STAT1 in macrophages (LysM-expressing) and/or conventional dendritic cells (CD11c-expressing) did not increase susceptibility to USUV.

A previous study from our group found that Vav-Cre/*Stat1*^fl/fl^ mice succumbed to lethal YFV-17D infection, yet Alb-Cre/*Stat1*^fl/fl^ and C57BL/6 mice exhibited 100% survival [60]. Thus, we have now demonstrated that STAT1 competency in hematopoietic cells specifically is required to protect against lethality following infection with two flaviviruses. Currently, it is not known whether STAT1 signaling competency is required in the same immune cell subsets to control YFV-17D and USUV infections; however, this could be explored through generation and infection of immune cell-specific STAT1 KO mice.

Our generation of LysM and CD11c-specific STAT1-deficient mice was inspired by a previous study, which found that mice lacking IFNAR1 in both of these compartments (LysM-CD11c-Cre/*Ifnar1*^fl/fl^) phenocopied full body *Ifnar1*^-/-^ mice, succumbing to lethal DENV infection by 5 dpi. Notably, mice lacking IFNAR1 in either the myeloid subset (LysM-Cre/*Ifnar1*^fl/fl^) or dendritic cells (CD11c-Cre/*Ifnar1*^fl/fl^) were also susceptible to DENV infection, but often started to recover once the the CD8 + T cell response was initiated around 5 dpi [78]. In this study, LysM-Cre/*Stat1*^fl/fl^, CD11c-Cre/*Stat1*^fl/fl^, and LysM-CD11c-Cre/*Stat1*^fl/fl^ mice were all resistant to USUV infection. This suggests that the affected immune cells may not be required for restricting USUV infection and perhaps different immune subsets are required for controlling USUV compared to DENV infection. Alternatively, the remaining STAT1-competent immune cells in LysM-Cre/*Stat1*^fl/fl^, CD11c-Cre/*Stat1*^fl/fl^, and LysM-CD11c-Cre/*Stat1*^fl/fl^ mice may be able to compensate for the loss of STAT1-mediated antiviral signaling to restrict USUV, but similar compensation of STAT1-competent immune cells in LysM-CD11c-Cre/*Ifnar1*^fl/fl^ may not be sufficient to restrict DENV. Future studies will have to focus on determining the specific immune subset(s) in which IFN signaling competency is essential for organism survival.

The mechanisms by which USUV establishes initial infection and spreads within a susceptible host remains poorly characterized. Understanding the immune system components that this virus interacts with or evades is essential for developing strategies to restrict infection. Here, we found that the absence of STAT1-mediated IFN signaling in hematopoietic cells allows USUV to overcome murine host defenses. We hypothesize that USUV infects certain immune cells, then exploits them as viral amplification reservoirs and/or shuttles to gain entry to peripheral tissues in a “Trojan horse” fashion. In these tissues, USUV replicates to high levels, thereby causing significant inflammation and damage. In future investigations, we aim to identify if specific immune cells become enriched during early stages of infection and how the cytokine response may accompany this. As USUV is an understudied virus with limited available reagents, this could be facilitated by the development of novel reporter viruses to mark infection *in vitro* and track infection *in vivo*.

## Supporting information

S1 Fig Histological analysis of peripheral tissues from USUV-infected mice. H&E staining of liver, spleen, kidney, lung, and brain tissues harvested from *Stat1*^fl/fl^, *Stat1*^-/-^, Alb-Cre/*Stat1*^fl/fl^, Syn-Cre/*Stat1*^fl/fl^, Vav-Cre/*Stat1*^fl/fl^ mice (n = 3/group) that were infected with 10^3^ FFU of USUV via subcutaneous (SC) injection or corresponding mock mice. For each mouse genotype, one representative image was chosen. The scale bars for liver, spleen, kidney, and brain images are 200 µm. The scale bar for lung images is 100 µm.
(PDF)

S2 Fig Infiltrating histiocyte-like immune cells in liver tissue are positive for USUV infection. Livers were harvested from *Stat1*^-/-^ mice that were infected with 10^3^ FFU of USUV via subcutaneous (SC) injection. Immunostaining of mouse liver tissue using the pan-immune cell marker CD45, the histiocyte marker CD68, anti-JEV NS3 to mark USUV infection, and DAPI. The scale bar indicates 50 µm.
(PDF)
