# Supplementary figures and images for: STAT1-mediated interferon signaling in the hematopoietic system is essential for restricting Usutu virus infection in vivo
Source: PLoS Negl Trop Dis. 2025 Jul 22;19(7):e0013317. doi: 10.1371/journal.pntd.0013317 (PMC12282914; doi:10.1371/journal.pntd.0013317)

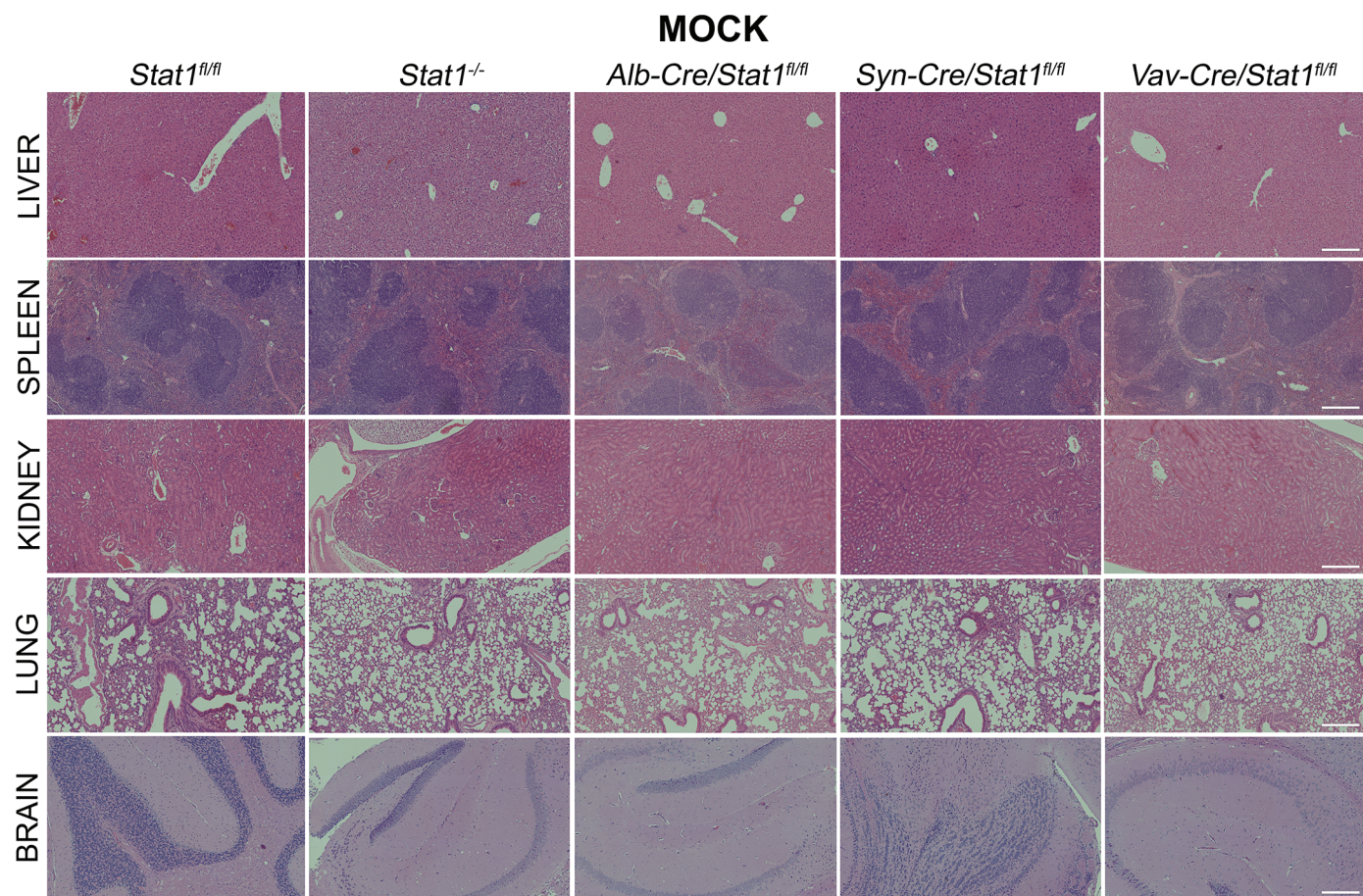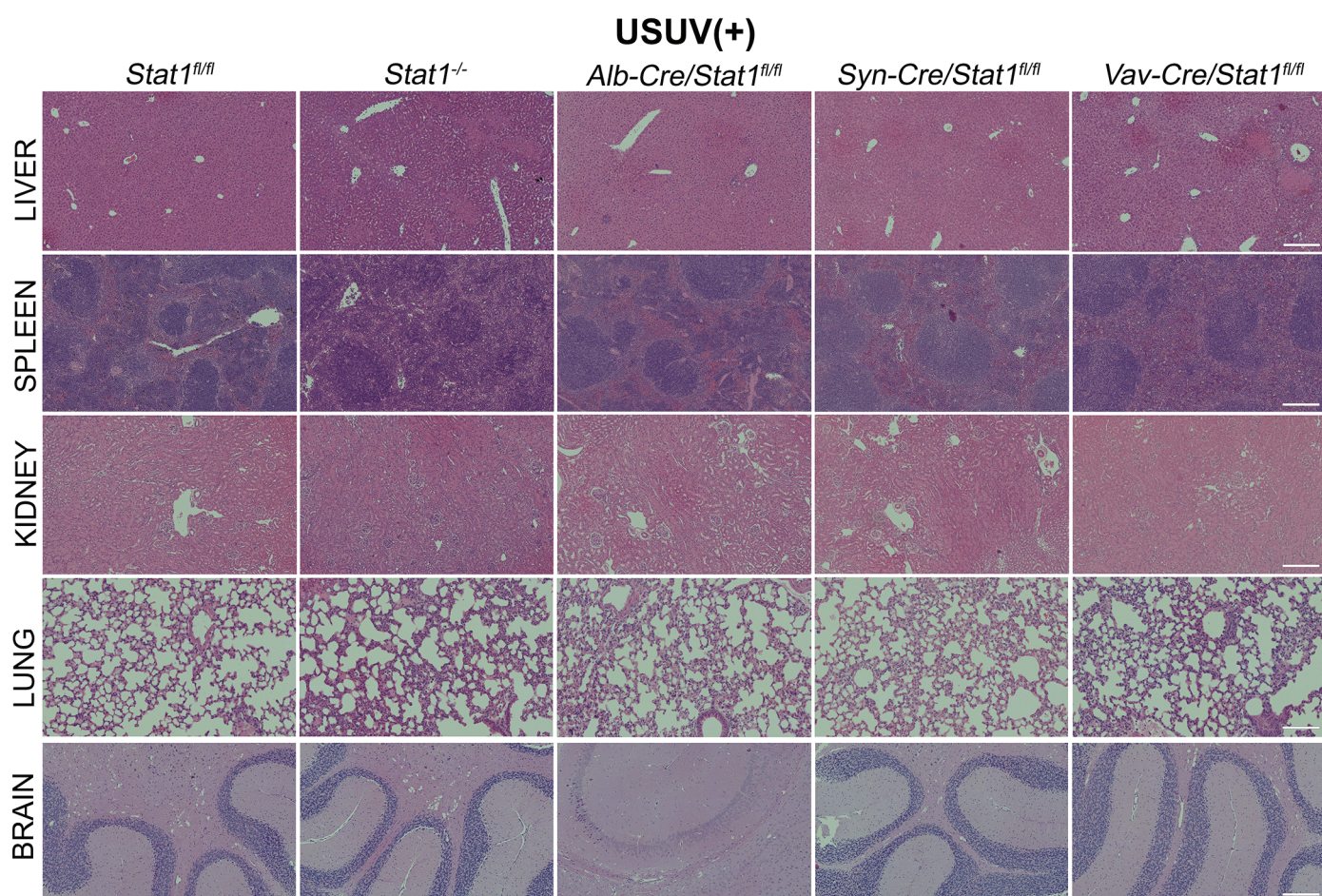

**S1 Fig**

Supplement: S1 Fig — H&E staining of liver, spleen, kidney, lung, and brain tissues harvested from Stat1fl/fl, Stat1-/-, Alb-Cre/Stat1fl/fl, Syn-Cre/Stat1fl/fl, Vav-Cre/Stat1fl/fl mice (n = 3/group) that were infected with 103 FFU of USUV via subcutaneous (SC) injection or corresponding mock mice. For each mouse genotype, one representative image was chosen. The scale bars for liver, spleen, kidney, and brain images are 200 µm. The scale bar for lung images is 100 µm. (PDF) [file pntd.0013317.s001.pdf]

MOCK

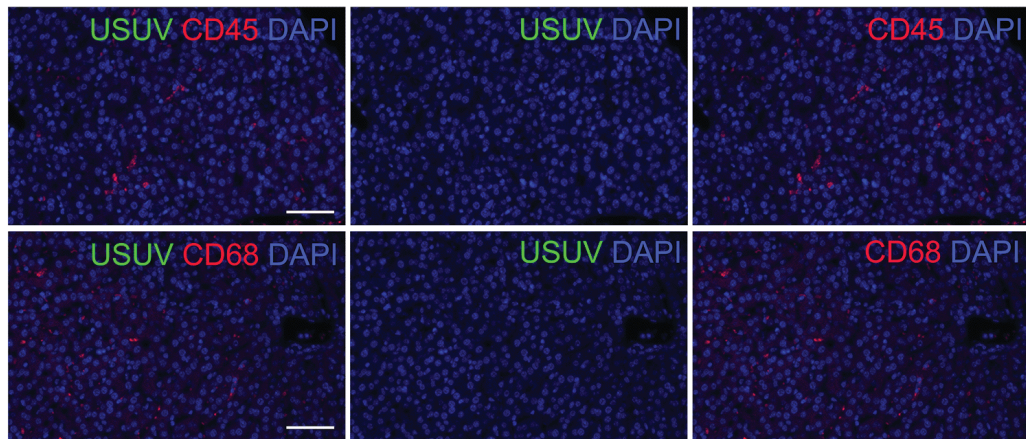

USUV(+)

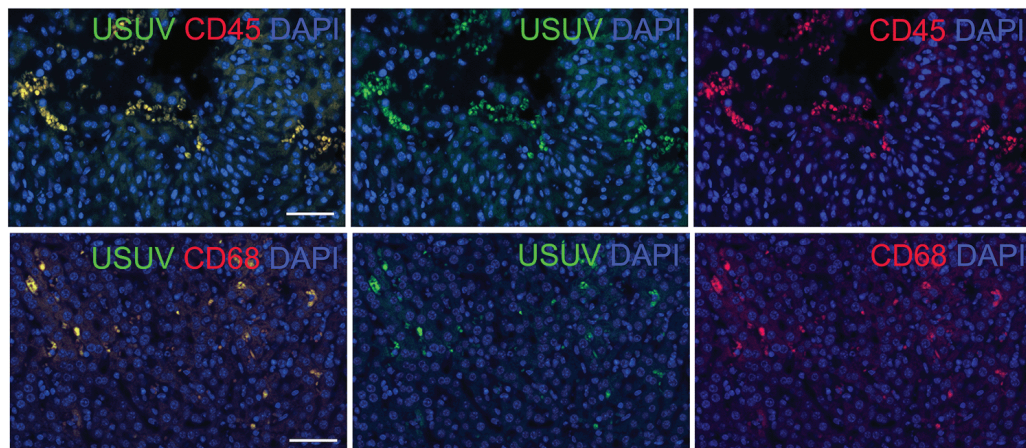

S2 Fig

Supplement: S2 Fig — Livers were harvested from Stat1-/- mice that were infected with 103 FFU of USUV via subcutaneous (SC) injection. Immunostaining of mouse liver tissue using the pan-immune cell marker CD45, the histiocyte marker CD68, anti-JEV NS3 to mark USUV infection, and DAPI. The scale bar indicates 50 µm. (PDF) [file pntd.0013317.s002.pdf]
